# Genetic inhibition of an ATP synthase subunit extends lifespan in *C. elegans*

**DOI:** 10.1038/s41598-018-32025-w

**Published:** 2018-10-04

**Authors:** Chen Xu, Wooseon Hwang, Dae-Eun Jeong, Youngjae Ryu, Chang Man Ha, Seung-Jae V. Lee, Lulu Liu, Zhi Ming He

**Affiliations:** 1 State Key Laboratory of Pharmaceutical Biotechnology, School of Life Sciences, Nanjing University, Nanjing, 210023 China; 2 Department of Life Sciences, Pohang University of Science and Technology, Pohang, Gyeongbuk 37673 South Korea; 3 Department of IT Convergence and Engineering, Pohang University of Science and Technology, Pohang, Gyeongbuk 37673 South Korea; 4 School of Interdisciplinary Bioscience and Bioengineering, Pohang University of Science and Technology, Pohang, Gyeongbuk 37673 South Korea; 5 Brain Research Core Facilities, Korea Brain Research Institute, Daegu, 41068 South Korea; 6 Present Address: Department of Pathology, Stanford University School of Medicine, Stanford, California 94305 USA

## Abstract

Mild inhibition of mitochondrial respiration leads to longevity. Disruption of mitochondrial respiratory components extends lifespan in *Caenorhabditis elegans*, but the effects appear to be complex and the underlying mechanism for lifespan regulation by mitochondrial respiratory genes is still not fully understood. Here, we investigated the role of Y82E9BR.3, a worm homolog of the ATP synthase subunit C, in modulating longevity in *C. elegans*. We found that the Y82E9BR.3 protein is localized in mitochondria and expressed in various tissues throughout development. RNAi knockdown of Y82E9BR.3 extends lifespan, decreases the accumulation of lipofuscin, and affects various physiological processes, including development delay, reproduction impairment and slow behavior. Further tissue-specific RNAi analysis showed that the intestine is a crucial organ for the longevity effects conferred by Y82E9BR.3 RNAi. Moreover, we demonstrated that lifespan extension by Y82E9BR.3 RNAi is associated with reduced mitochondrial function, as well as the suppression of complex I activity in mitochondria. Unexpectedly, Y82E9BR.3 RNAi knock down did not influence the whole-worm ATP level. Our findings first reveal the crucial role of Y82E9BR.3 in mitochondrial function and the underlying mechanism of how Y82E9BR.3 regulates lifespan in *C. elegans*.

## Introduction

Mitochondria play an essential role in many important physiological processes, including aging. Mitochondrial function has been thought to gradually decline with age, while oxidative damage and mitochondrial DNA mutations accumulate^1,2^. Although complete disruption of mitochondrial function is detrimental or even lethal for many eukaryotes, including humans, accumulating evidence has revealed that partial inhibition of mitochondrial function tends to increase lifespan^3–5^. In *C. elegans*, mutations in various mitochondrial electron transport chain (ETC) genes can greatly extend lifespan; these include mutations in *isp-1* (encoding the iron sulfur protein of Complex III)^6^ and *clk-1* (encoding the hydroxylase protein necessary for the biosynthesis of the ETC electron transporter coenzyme Q)^7,8^. In addition, RNAi knockdown of various mitochondrial ETC genes prolongs lifespan in yeast, worms and fruit flies^9–11^.

The effects of mitochondrial ETC genes on modulating lifespan appear to be complex. Inhibition of some ETC genes increases lifespan, whereas inhibition of others decreases or does not alter lifespan in *C. elegans* and *Drosophila*. For example, mutations in *mev-1*, which encodes a subunit of the succinate dehydrogenase cytochrome *b* of complex II, causes a short lifespan in worms^12^. In addition, the underlying causes for lifespan regulation by ETC genes remain incompletely understood. For example, the roles of reactive oxygen species (ROS) production and mitochondrial function in aging and lifespan of ETC mutants can be opposite ways^3,4^. One model interpreting these opposite effects is that moderate mitochondrial impairments increase lifespan until a threshold is reached, beyond which animals display wide-spread damage, shortened lifespan, or even death^3,4,13^. Nevertheless, how mitochondrial genes modulate lifespan and whether they function in modulating lifespan in other species remain incompletely elucidated.

ATP synthase, also known as complex V of the mitochondrial respiratory chain, is the primary cellular energy-generating machinery^14,15^. ATP synthase is composed of a proton channel F_0_ and a peripheral catalytic domain F_1_. Proton transport through the F_0_ domain drives the release of ATP product on F_1_ through long-range conformational changes^16^. In mammals, ATP synthase deficiency is one of the rarer mitochondrial oxidative phosphorylation deficiencies, covering only 1%^16^. Selective deficiency of the human mitochondrial ATP synthase of nuclear origin caused by altered biosynthesis of the enzyme results in heart failure^17^.

ATP synthase is also intimately linked to aging. In worms, genetic inhibition of the *atp-2* gene, which encodes the beta-subunit of F_1_ domain in complex V, leads to developmental delay and increased lifespan^18^. Additionally, a genome-wide RNAi screen revealed that RNAi knockdown of *atp-3* (encoding O subunit of F_1_ domain), *atp-4* (encoding ATP synthase coupling factor 6 of F_0_ domain), *atp-5* (encoding ATP synthase D chain of F_0_ domain) or *asb-2* (encoding ATP synthase B homolog of F_0_ domain) prolongs worm lifespan^19,20^. However, the underlying mechanism for lifespan extension due to inhibition of these subunits in the ATP synthase remains unclear. As ATP synthase is highly conserved throughout evolution^14,15^, understanding the role of the ATP synthase in lifespan regulation can lead to untangling of the complexity of mitochondrial ETC genes in modulating lifespan.

*C. elegans* Y82E9BR.3 is a worm homolog of the ATP synthase subunit C. We previously reported that knockdown of Y82E9BR.3 using RNA interference promoted longevity in worms^21^, which is also observed in another report^22^. However, the role of Y82E9BR.3 in mitochondrial function and lifespan regulation remained uncharacterized. Here, we found that the Y82E9BR.3 protein is localized in mitochondria and expressed in various tissues throughout development. Genetic inhibition of Y82E9BR.3 lengthens lifespan associated with reduced mitochondrial function as well as a decrease in complex I activity in mitochondria. In addition, the intestine proved a crucial organ for the longevity effects conferred by Y82E9BR.3 RNAi. Collectively, our study demonstrated the important role of Y82E9BR.3 in mitochondrial function and the underlying mechanism involving Y82E9BR.3 in aging.

## Results

### Y82E9BR.3 RNAi extends lifespan and decreases lipofuscin accumulation

**Figure 1:**
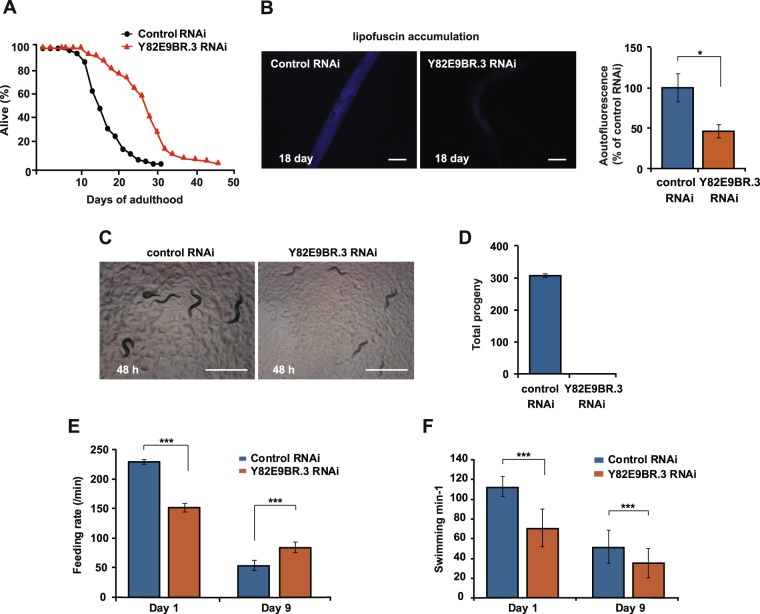
Y82E9BR.3 RNAi-treated *C. elegans* displays various physiological phenotypes. (**A**) Y82E9BR.3 RNAi extended the lifespan of wild-type animals. See Supplementary Table S1 for statistical analysis and additional repeats. (**B**) Representative images showing Y82E9BR.3 RNAi reduced lipofuscin accumulation in aged wild-type animals. Scale bar indicates 50 μm. Quantification of fluorescence on the left by using ImageJ. The graph depicts the mean percentage in arbitrary units relative to that of control RNAi-treated worms on day 18 (n ≥ 20 from three independent experiments). Error bars represent SEM (*P < 0.05, two-tailed Student’s *t*-test). (**C**) Y82E9BR.3 RNAi delayed the development of wild-type animals. Scale bar indicates 1 mm. (**D**) Y82E9BR.3 RNAi caused complete sterility in wild-type animals. (n = 10 from duplicate independent experiments). Error bars represent SEM. (**E**) Knockdown of Y82E9BR.3 reduced the feeding rate (number of pumps per minute) of wild-type animals in young (Day 1) worms and delayed the age-dependent decline of feeding in old (Day 9) worms. (n = 20 from duplicate independent experiments). Error bars represent SEM. (***P < 0.001, two-tailed Student’s *t*-test). (**F**) Knockdown of Y82E9BR.3 reduced the swimming rate (frequency of swimming) of wild-type animals in young (Day 1) and old (Day 9) worms. (n = 30 from duplicate independent experiments). Error bars represent SEM. (***P < 0.001, two-tailed Student’s *t*-test).

To understand the role of Y82E9BR.3 in mitochondrial function and aging, we knocked down the gene using RNA interference. We found that Y82E9BR.3 expression was significantly reduced by approximately 90% using the RNAi clone in worms and Y82E9BR.3 RNAi effectively decreased Y82E9BR.3::GFP levels (Fig. S1A–C), and aged animals displayed slight reduction in Y82E9BR.3 mRNA expression compared to young worms (Fig. S1D), showing Y82E9BR.3 expression was largely unaffected with age. We then determined the effect of Y82E9BR.3 RNAi on the lifespan of worms. We found that Y82E9BR.3 RNAi significantly extended lifespan by 60% (Fig. 1A). It is believed that mitochondria contribute to the lipofuscin accumulation with age, such that the mitochondrial ATP synthase subunit c was found a major component in lipofuscin formed under pathological conditions^23,24^. We found that the accumulation of lipofuscin was remarkably decreased by Y82E9BR.3 RNAi (Fig. 1B). Thus, knockdown of Y82E9BR.3 delays aging and extends lifespan in *C. elegans*.

### Y82E9BR.3 RNAi affects various physiological processes

We further examined whether knockdown of Y82E9BR.3 affected other physiological processes. First we asked whether Y82E9BR.3 RNAi affected larval development. Compared to control RNAi, Y82E9BR.3 RNAi-treated worms required considerably longer time to develop from hatching to adulthood (approximately 4 days). Y82E9BR.3 RNAi-treated worms exhibited a smaller body size than the wild type 48 h after hatching (Fig. 1C).We also found that Y82E9BR.3 RNAi resulted in complete sterility (Fig. 1D). We next determined the behavioral phenotypes of Y82E9BR.3 RNAi-treated *C. elegans*. Feeding rates reflect food intake rates in worms. We found that Y82E9BR.3 RNAi treated worms showed a reduced feeding rate in young worms, whereas a higher feeding rate in old worms compared to the control (*p* < 0.001) (Fig. 1E), exhibiting a slower rate of decline in feeding than the control. We employed the swimming rate assay, a well-established behavior assessment that evaluates the motility of worms. We found that Y82E9BR.3 RNAi-treated worms showed a decreased swimming rate in both young and old worms compared to the control (*p* < 0.001) (Fig. 1F), exhibiting a similar rate of decline in swimming with the control. Thus, knockdown of Y82E9BR.3 delays development, impairs reproduction and slows behavior in *C. elegans*.

### Mitochondrial Y82E9BR.3 is expressed in various tissues throughout development

**Figure 2:**
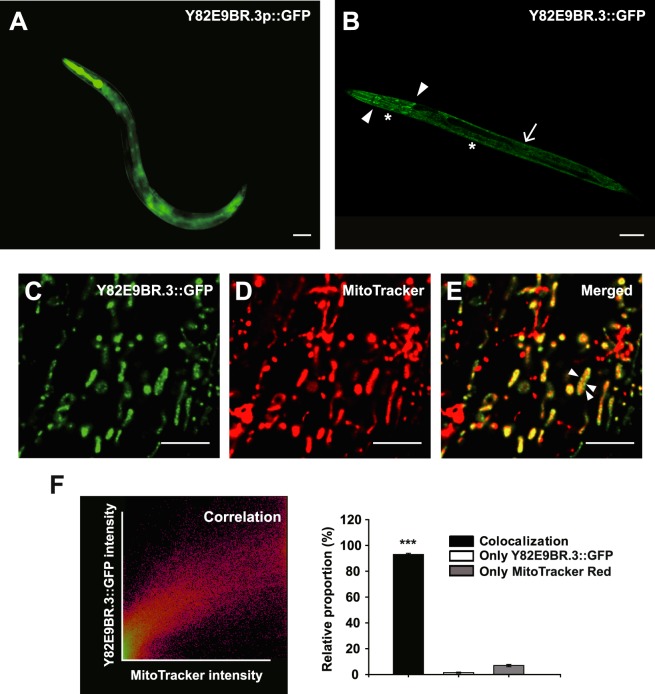
Expression pattern and localization of Y82E9BR.3 in transgenic animals. (**A**) GFP expression in *Y82E9BR.3p::gfp* transgenic animals. GFP driven by a Y82E9BR.3 promoter was strongly expressed in pharyngeal muscles and widely distributed in the whole body, including the intestine, body wall muscles and hypodermis in an adult animal. Scale bar indicates 50 μm. (**B**) Expression patterns of Y82E9BR.3 in multiple tissues of *Y82E9BR.3::gfp* transgenic animals. *Y82E9BR.3p::Y82E9BR.3::gfp* was predominantly expressed in pharyngeal muscles (arrowhead), intestine (arrow) and hypodermis (asterisk). Scale bar indicates 75 μm. Worms at the L2 or L3 larval stage were used for these images. (**C**–**E**) Images of an L2 larval animal expressing Y82E9BR.3::GFP driven by a Y82E9BR.3 promoter. Y82E9BR.3::GFP (**C**) and MitoTracker that stained mitochondria (**D**) were colocalized. (**E**) The fluorescent pattern of Y82E9BR.3::GFP highly overlapped with mitochondria and was substantially colocalized with the patterns on mitochondrial membrane (arrowhead). Scale bars indicate 5 μm. (**F**) Quantification of colocalizaton of Y82E9BR.3::GFP and Mitotracker. 2D intensity histogram output of colocalization analysis performed with NIS software (*left*). The Pearson coefficient of the pixel-intensity correlation is 0.83 in panel (C–E) as well as the percentage of colocalization coefficient is compared to green image 88%, red image 96% in the combination area. To ensure accuracy, the number of colocalization between Y82E9BR.3::GFP and MitoTracker in the 20 square micrometer were counted (*right*). The Y82E9BR.3::GFP was 98% colocalized with MitoTracker-stained mitochondria (n = 10). Error bars represent STD (***P < 0.001, two-tailed Student’s *t*-test).

We then visualized tissues that expressed Y82E9BR.3 by generating and examining a Y82E9BR.3 promoter-driven GFP-expressing transgenic animal (*Y82E9BR.3p::gfp*). We found that the strongest fluorescence was observed in the pharyngeal muscles, and the signal was widely distributed in the whole body of adult animals, including the intestine, body wall muscles and hypodermis (Fig. 2A). *Y82E9BR.3p::gfp* was expressed in all developmental stages, including eggs and larvae (Data not shown).

To further examine the cellular and intracellular distribution and localization of Y82E9BR.3 protein in *C. elegans*, we generated transgenic animals that expressed *Y82E9BR.3::gfp* fusion protein under its own promoter (*Y82E9BR.3p::Y82E9BR.3::gfp*). *Y82E9BR.3::gfp* was predominantly expressed in the pharyngeal muscle, intestine and more faintly in the hypodermis (Fig. 2B). We then determined the subcellular localization of Y82E9BR.3 protein with MitoTracker Red, a fluorescent marker of mitochondria. GFP-fused Y82E9BR.3 protein was present in a pattern typical of hypodermal mitochondria (Fig. 2C–F). The staining of MitoTracker Red exhibited a web-like, cytoplasmic staining pattern, highly similar to the GFP expression pattern. These results indicate that Y82E9BR.3 proteins are localized in mitochondria.

### Y82E9BR.3 in the intestine plays a key role in longevity

**Figure 3:**
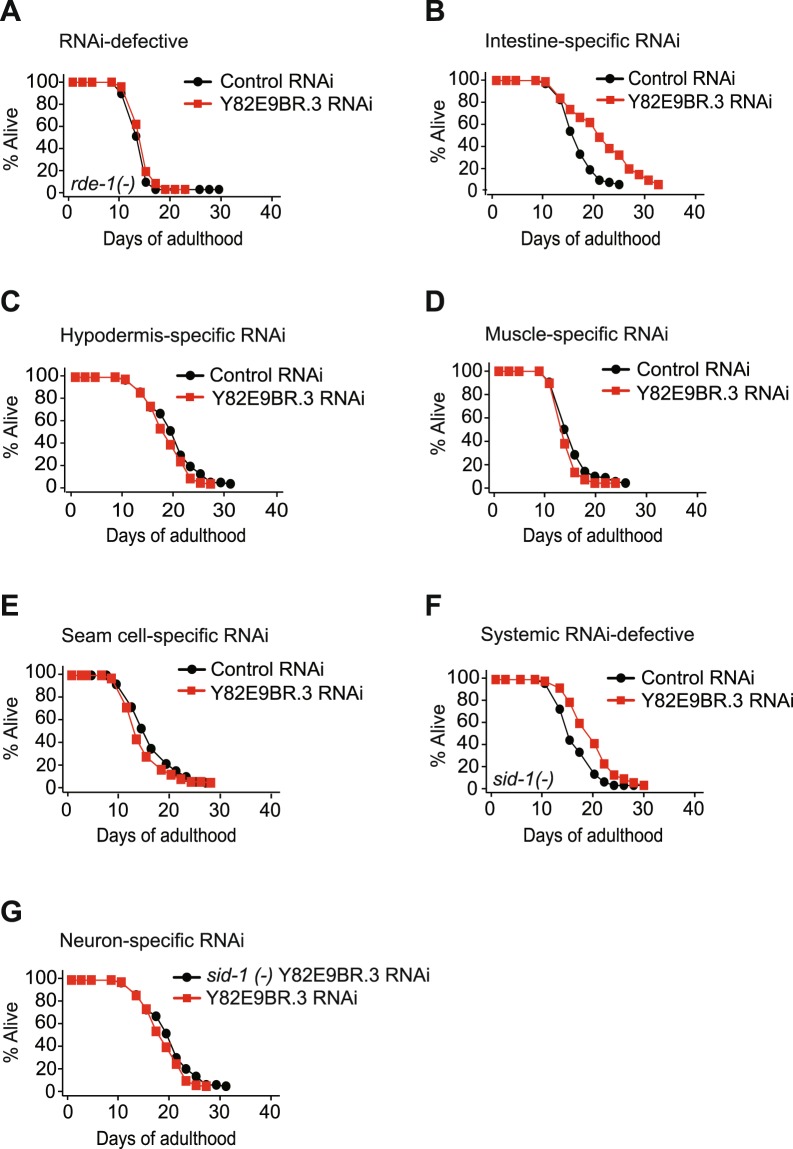
Role of Y82E9BR.3 for longevity in various tissues. (**A**) Y82E9BR.3 RNAi did not influence the lifespan of *rde-1(ne219)* mutants. (**B**) Intestine-specific (*rde-1(ne219); kbls7[nhx-2p::rde-1; rol-6D]*) Y82E9BR.3 RNAi increased the lifespan of animals. (**C**) Hypodermis-specific (*rde-1(ne219); kzls9[lin-26p::nls::gfp; lin-26p::rde-1; rol-6D]*) Y82E9BR.3 RNAi did not influence the lifespan of animals. (**D**) Y82E9BR.3 RNAi knockdown specifically in muscles (*rde-1(ne219); kzls20[hlh-1p::rde-1; sur-5p::nls::gfp]*) did not influence the lifespan of animals. (**E**) Y82E9BR.3 RNAi knockdown specifically in seam cells (*rde-1(ne219); Is[wrt-2::rde-1]*) did not influence the lifespan of animals. (**F**) Y82E9BR.3 RNAi slightly increased lifespan in RNAi-defective *sid-1(pk3321)* mutants. (**G**) Neuron-specific (*sid-1(pk3321); uIs69[myo-2p::mCherry; unc-119p::sid-1]*) Y82E9BR.3 RNAi did not live longer than *sid-1(−)* mutants treated with Y82E9BR.3 RNAi. See Supplementary Table S1 for statistical analysis and additional repeats for survival and lifespan data shown in this figure.

To examine the tissues in which Y82E9BR.3 contributed to longevity induced by Y82E9BR.3 RNAi, we performed lifespan assays using tissue-specific RNAi methods. We first confirmed that Y82E9BR.3 RNAi did not influence lifespan in an RNAi-defective *rde-1* mutant background (Fig. 3A). We found that intestine-specific Y82E9BR.3 RNAi significantly increased lifespan by 29% (Fig. 3B). In contrast, Y82E9BR.3 RNAi specific for hypodermis, muscles, or seam cells did not influence lifespan (Fig. 3C–E). As neurons are refractory to RNAi^25^, we used the *sid-1* transgenic system for neuron-specific RNAi experiments^26^. We found that Y82E9BR.3 RNAi slightly, but significantly, increased lifespan in systemic RNAi-defective *sid-1* mutants (Fig. 3F). As feeding RNAi operates in the intestinal cells of *sid-1* mutants, this is consistent with our data regarding extended lifespan via the intestine-specific Y82E9BR.3 RNAi (Fig. 3B). We then showed that neuron-specific Y82E9BR.3 RNAi animals did not live longer than *sid-1* mutants treated with Y82E9BR.3 RNAi (Fig. 3G). Together, the data indicate that the intestine appears to be a crucial organ for the longevity of Y82E9BR.3 RNAi-treated *C. elegans*.

The result was further substantiated by the finding that Y82E9BR.3 RNAi remarkably decreased Y82E9BR.3 protein levels in various tissues including the intestine (Fig. S1B,C) when Y82E9BR.3 RNAi was applied in Y82E9BR.3p::Y82E9BR.3::GFP transgenic animals.

### Y82E9BR.3 RNAi reduces mitochondrial respiration

**Figure 4:**
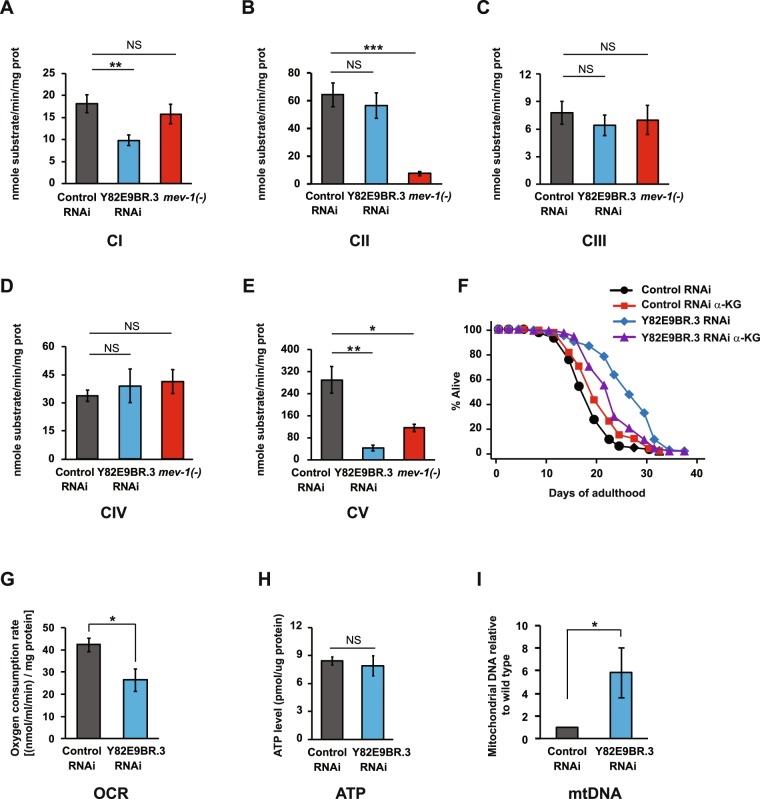
Effect of Y82E9BR.3 RNAi on mitochondrial function. (**A**–**E**) Effects of Y82E9BR.3 RNAi on mitochondrial respiratory chain (MRC) enzymatic activities: rotenone-sensitive NADH-decylubiquinone oxidoreductase (CI) (**A**); succinate dehydrogenase (CII) (**B**) antimycin A-sensitive decylubiquinol cytochrome c oxidoreductase (CIII) (**C**); cytochrome c oxidase (CIV) (**D**) and ATP synthase (CV) (**E**). MRC enzymatic activities were determined spectrophotometrically with mitochondria isolated from worms. Data are represented as the mean ± SEM (n = 5 for *mev-1(−)* group, n = 8 for control and Y82E9BR.3 RNAi groups). *P < 0.05, **P < 0.01, two-way ANOVA test. (**F**) Lifespan of wild-type worms treated with Y82E9BR.3 RNAi with or without 8 mM α-ketoglutarate (α-KG). See Supplementary Table S1 for statistical analysis and additional repeats. (**G**) Oxygen consumption rates were significantly decreased by Y82E9BR.3 RNAi. Oxygen consumption rate of wild-type worms treated with Y82E9BR.3 RNAi or control RNAi was measured by a Clark electrode. Values were normalized to total protein levels (means ± SEM, n = 5 independent experiments, *P < 0.05 calculated from two-tailed Student’s *t*-test). (**H**) Y82E9BR.3 RNAi did not change ATP levels. ATP levels were measured in wild-type worms treated with Y82E9BR.3 RNAi or control RNAi using a luciferase-based assay. Values were normalized to total protein levels (means ± SEM, n = 6 from three independent experiments, two-tailed Student’s *t*-test). (**I**) Y82E9BR.3 RNAi increased the mitochondrial DNA contents. Percent increase in total mitochondrial DNA relative to wild type is shown, as measured by qPCR. Data are presented from seven independent biological repeats. Error bars represent SEM (*P < 0.05; two-tailed Student’s *t*-test).

To determine whether knockdown of Y82E9BR.3 affected mitochondrial function, we measured the activities of individual components of the mitochondrial respiratory chain (MRC) in Y82E9BR.3 RNAi-treated animals. We also used short-lived *mev-1* mutants as a positive control. We found that Y82E9BR.3 RNAi significantly decreased the activity of complex I (rotenone-sensitive NADH ubiquinone oxidoreductase) (*p* < 0.01, Fig. 4A). In contrast, *mev-1* mutants did not show a significant change in complex I activity (Fig. 4A). We found that the complex II activity of Y82E9BR.3 RNAi treated-worms was not significantly reduced (Fig. 4B). Consistent with previous reports^12^, *mev-1* mutants exhibited a significant decrease in complex II (succinate dehydrogenase) activity (*p* < 0.05, Fig. 4B). We showed that the activity of complex III (antimycin A-sensitive decylubiquinol cytochrome c oxidoreductase) or complex IV (cytochrome c oxidase) did not significantly decrease in Y82E9BR.3 RNAi-treated worms or in *mev-1* mutants (Fig. 4C,D). Complex V (ATP synthase) activity significantly decreased in Y82E9BR.3 RNAi-treated worms and in *mev-1* mutants (*p* < 0.05, Fig. 4E). Together, these data suggest that Y82E9BR.3 RNAi significantly decreases the activity of complex I, as well as ATP synthase activity.

The Krebs cycle metabolite α-ketoglutarate (α-KG) promotes longevity in *C. elegans* by inhibiting complex V, ATP synthase^27^. As we showed that Y82E9BR.3 RNAi reduced the activity of complex V (Fig. 4E), we wondered if Y82E9BR.3 RNAi and α-KG acted together to affect lifespan. We found that α-KG treatment extended the lifespan of wild-type worms by 11%, without further increasing longevity in Y82E9BR.3 RNAi-treated worms, but actually decreased the lifespan of Y82E9BR.3 RNAi-treated worms (Fig. 4F). These data are consistent with our data showing that Y82E9BR.3 regulates lifespan by affecting MRC.

To examine the effects of Y82E9BR.3 RNAi on the MRC components in animal physiology, we measured the oxygen consumption rates and ATP production using whole animals. We found that oxygen consumption rates were significantly reduced in intact young adult worms treated with Y82E9BR.3 RNAi by 37.5% compared to the control (*p* < 0.05, Fig. 4G). We then measured overall ATP production in animals. Surprisingly, we found that Y82E9BR.3 RNAi-treated worms did not display decreased ATP levels (Fig. 4H). These data suggest that Y82E9BR.3 RNAi reduces mitochondrial respiration activities, leading to a response that compensates the low levels of ATP synthesis.

In addition, we found Y82E9BR.3 RNAi increased the mitochondrial DNA contents (Fig. 4I) as other mitochondrial component mutations do^28^.

## Discussion

The effect of mitochondrial ETC genes on lifespan is complicated, and the regulatory mechanism remains incompletely understood. Here, we investigated how the ATP synthase subunit C worm homolog Y82E9BR.3 influences the mitochondrial function in aging. We have shown that Y82E9BR.3 knockdown extends lifespan, decreases lipofuscin accumulation, and affects various physiological processes. The Y82E9BR.3 protein is localized in mitochondria and is expressed in various tissues throughout development. Intestine-specific Y82E9BR.3 RNAi is sufficient for lifespan extension. Furthermore, we have demonstrated that lifespan extension induced by Y82E9BR.3 knockdown is associated with reduced mitochondrial function as well as reduction of complex I activity. Overall, our findings reveal that mitochondrial gene Y82E9BR.3 plays an important role in determining aging and lifespan in *C. elegans*, which represents the complexity between mitochondrial function and aging.

Mild inhibition of mitochondrial respiration leads to longevity in many organisms and genes expressed in the mitochondria represent the largest group of genes that can affect lifespan. Our data showed that Y82E9BR.3 knockdown extends lifespan and decreases the lipofuscin accumulation, in agreement with the lifespan extension effect of *atp-2*, *atp-3*, *atp-4*, *atp-5* and *asb-2* encoding the different subunits of ATP synthase^19,20^. Using an RNAi dilution approach, a threshold effect between mitochondrial ETC gene disruption and life extension was determined^29^. The *atp-3* showed a typical three-phase lifespan response^29^: when *atp-3* mRNA levels were reduced to approximately 80% of a normal level, mean lifespan began to increase; when *atp-3* mRNA levels further decrease to below 40% of a normal level, mean lifespan was greatly reduced. In contrast, other ETC genes such as *cco-1*, *isp-1* and *nuo-2*, do not have a bell-shaped lifespan curve: RNAi disruption of these genes results in an increasing lifespan response with increasing concentrations of RNAi bacteria^30^. In our study, when Y82E9BR.3 mRNA was lowered to approximately 10% of a normal level, worms displayed an extended lifespan by approximately 60%. Thus, the lifespan response of Y82E9BR.3 is more likely to be similar to the case of *nuo-2*, rather than exhibiting a three-phase lifespan response. Additionally, FUdR has been shown to impact mitochondrial function^31^ and extend the lifespan of some mutant worms^32^. To minimize the effect of FUdR on lifespan assay, we used relatively low (5 µM) concentrations of FUdR, which is much lower than the concentration of FUdR previously reported to have effects on mitochondria function. However, we still cannot completely exclude the possibility that FUdR might have affected mitochondrial function. Importantly, we found that the extended lifespan of Y82E9BR.3 RNAi-treated animals without FUdR was similar to that performed with FUdR (Fig. S2).

In addition to life extension, Y82E9BR.3 RNAi-treated worms exhibited a typical “Mit phenotype”, including a development delay, small adult body size, altered brood size and slow behavior^33^, which is also observed in another report^22^. Compared to *isp-1*, *gas-1* and *clk-1* mutants that display a reduced brood size^34,35^, Y82E9BR.3 knockdown worms were completely sterile. This might be because disruption of Y82E9BR.3 greatly reduces ATP synthesis, and worms defective in Y82E9BR.3 cannot provide sufficient energy for the energy-intensive process of germ-line proliferation and organogenesis^18,36^, resulting in complete sterility. However, the function of ATP synthase in Y82E9BR.3 RNAi worms was not completely destroyed and the energy was able to support worm development from the larval to adult stage. The impaired mitochondrial function delays larval development and slows adult motility, both of which likely require a high rate of respiration during proliferation and in muscle cells. Our data are consistent with data for *atp-2*, which encodes subunit b of ATP synthase. Defects in *atp-2* result in developmental arrest at the third larval stage and impaired pharyngeal pumping and defecation, as well as a lengthened lifespan^18^. Comparatively, ATPsyn-d (a homolog of *atp-5* in *C. elegans*, encoding ATP synthase D chain of F0 domain) knockdown prolongs lifespan, but does not affect locomotor activity in *Drosophila*^37^. This might be attributable to differences in gene expression in various tissues, and the integrated response from these tissues represents the final phenotype. Notably, swimming and feeding in Y82E9BR.3 RNAi-treated worms decline with age in different manners, in accordance with a previous report^38^. This might be related to the difference of Y82E9BR.3 RNAi efficiency in different tissues.

It has been suggested that the RNAi targeting ETC genes in specific tissues is essential for establishing and maintaining a prolongevity cue^39^. In our study, Y82E9BR.3 is expressed in tissues of pharynx, hypodermis and intestine, and Y82E9BR.3 RNAi successfully knocked down the Y82E9BR.3 expression in all these tissues. The tissue-specific RNAi lifespan assay further showed that among those tissues, the intestine was required for the lifespan regulation. Our finding is in line with the study of the *cco-1* subunit of complex IV in which intestinal- or neuronal-specific RNAi knockdown prolongs worm lifespan^39^. Our data further support the notion that key tissues transmit longevity signals to modulate aging processes in a whole organism. Here, long lifespan conferred by intestine-specific Y82E9BR.3 RNAi is shorter than that of Y82E9BR.3 RNAi in wild-type worms, which is probably related to the possibilities that the genetic backgrounds of those strains are different and lifespan results vary among sets. In addition, our data also suggest that Y82E9BR.3 expression does not change with age.

Genetic inhibition of mitochondrial ETC genes both by mutation and RNAi prolongs lifespan in *C. elegans*, but the mechanism varies, even when targeting the same genes^40^. In our study, we found that Y82E9BR.3 knockdown significantly decreased the activity of complex I, as well as ATP synthase activity. This result suggests that the complex I-associated pathway is involved in the effects of ATP synthase subunit C on lifespan. The interdependence among the individual components of ETC has been reported previously. For example, long-lived *clk-1* mutants have a severely defective complex I-dependent metabolism due to differences in complexes I and II in their ability to use quinone pools^41^. In addition, long-lived *isp-1* mutants have a decreased complex I activity due to allosteric interaction between ETC components^42^. Complex IV knockdown shortens nematode lifespan, and decreases complex I enzymatic activity due to interdependence among individual components of the ETC supercomplex^43^. These findings show that defects in a single ETC component can elicit wide ranging effects on the function of various ETC complexes. Additionally, polyphenolic ATP synthases inhibitors have been shown to inhibit complex I activity, but did not significantly inhibit the activity of complexes II and III^44,45^, indirectly supporting our data. Therefore, we speculate that the disruption of the ATPsyn subunit C in the tightly coupled MRC would affect multiple enzymatic steps of electron transfer and may disturb proton gradient and electron carriers, resulting in reduced complex I activity. However, specific mechanisms still require further investigation.

We observed that the activity of complex III or complex IV was not significantly decreased in Y82E9BR.3 RNAi-treated worms. This result is in agreement with the effect of ATPsyn-d (*atp-5*), in which ATPsyn-d knockdown does not significantly alter the activities of complex III and complex IV or the formation of five oxidative phosphorylation complexes in *Drosophila*^37^.

The *mev-1* mutants, which we used as a positive control in mitochondrial enzymatic assays, exhibited decreased enzymatic activities for complex II and complex V, but normal activities for complex I, III and complex IV. This result is consistent with previous reports^46^, in which *mev-1* mutants are normal for NADH-cytochrome c oxidoreductase (complex I plus III) activity but have severely reduced succinate-cytochrome c oxidoreductase (complex II plus III) activity. Here, we report for the first time that complex V activity significantly decreased in *mev-1* mutants, in support of the notion of intimate interdependence among MRC components. Our data also suggest that ATP synthase might play a role in the aging phenotypes of *mev-1* mutants. However, ATP levels are normal in *mev-1* mutants^46^, further representing the complexity between mitochondrial function and aging.

In mitochondrial mutants or starved *C. elegans*, α-KG and other metabolic products with similar structures to α-KG are enriched^47^. Additionally, α-KG binds the b-subunit (ATPsyn-b, *atp-2*) of complex V and extends nematode lifespan^27^. In our study, we exogenously treated Y82E9BR.3 knockdown worms with α-KG, and did not observe an additive lifespan-increasing effect, supporting the notion that Y82E9BR.3 regulates lifespan by affecting MRC. Notably, α-KG decreased the lifespan of Y82E9BR.3 RNAi-treated worms. One possible explanation for this is the antioxidant effect of α-KG^48^. Long-lived mitochondrial respiration mutant *C. elegans* have been reported to contain higher levels of ROS than wild-type animals^49^. Recent studies demonstrated that the increased ROS levels promote longevity in mitochondrial respiration mutant *C. elegans* and antioxidant treatment abolishes the long lifespan caused by the inhibition of mitochondrial respiration^49–51^. Therefore, it seems likely that Y82E9BR.3 RNAi increased ROS levels, and this in turn probably promotes the longevity conferred by Y82E9BR.3 RNAi. α-KG may have counteracted the ROS and thereby abolish the long lifespan conferred by Y82E9BR.3 RNAi. This also suggests the potential role of ROS in the mechanism by which Y82E9BR.3 knockdown leads to longevity.

Although mutation or RNAi knockdown of the genes *atp-2, atp-3, atp-4, atp-5* and *asb-2*, which encode different subunits of ATP synthase, prolongs nematode lifespan^19,20^, the underlying mechanisms remain mostly uncharacterized. In particular, a recent study showed that knockdown of ATPsyn-d (*atp-5*) interacts with TOR signaling to modulate protein homeostasis and lifespan in *Drosophila*, showing a conserved role for ATP synthase in modulating lifespan^37^. Moreover, α-KG inhibits ATP synthase, likely by binding to ATPsyn-b (*atp-2*), and reduces TOR signaling to extend lifespan in *C. elegans*^27^. It seems likely that mTOR signaling plays an important role in the longevity induced by the inhibition of ATP synthase subunits. Inhibition of TOR signaling also extends lifespan in several model organisms and the longevity conferred by inhibition of TOR signaling is associated with mitochondrial activity^4^. For example, in *Drosophila*, dietary restriction upregulates 4E-BP, a translational repressor in TOR signaling, to increase the expression of genes essential for mitochondrial function, including mitochondrial complexes I and IV. Lifespan extension induced by dietary restriction is suppressed by knocking down the ETC genes regulated by 4E-BP^52^. In addition, TOR signaling increases mitochondrial respiration through YY1 (yinyang 1), a key mediator of the interaction between TOR and PGC-1α in mammalian cells^53^. These lines of evidence suggest a possible correlation between ATP synthase, TOR signaling and mitochondrial complex I in aging, indirectly supporting our finding. It further implies that the mTOR pathway might mediate ATPsyn-subunit C knockdown-induced longevity.

ATP generation can be achieved through the coupling of glycolysis to oxidative phosphorylation in mitochondria, which is oxygen dependent. Therefore, the oxygen consumption rate of an organism is an important indicator of normal cellular function. In our study, we used the Clark electrode instrument to monitor the continuous decrease in oxygen concentration using whole worms. In agreement with the idea that the disruption of the ETC component genes would result in altered patterns of oxygen consumption, Y82E9BR.3 knockdown reduced mitochondrial respiration. Surprisingly, Y82E9BR.3 knockdown did not alter ATP levels. One possibility might be the initiation of a compensatory response to low levels of ATP synthesis. In worms, a glyoxylate pathway serves as an alternative energy source that can bypass mitochondrial MRC dysfunction^54^. Alternatively, we assume that Y82E9BR.3 RNAi-treated worms might have decreased energy expenditure, thereby maintaining ATP at a normal level. In a previous report, deficiency in *clk-1* decreases mitochondrial function with normal or increased ATP levels, likely due to their decreased energy utilization^55^. Another possibility is glycolysis can be upregulated given that cellular levels of ATP are tightly regulated.

For other genes encoding ATPsyn subunits, *atp-2* knockdown decreases ATP level and oxygen consumption and prolongs lifespan in worms^27^, whereas ATPsyn-d (*atp-5*) knockdown does not significantly change or even increases ATP levels in *Drosophila*^37^. Apparently, Y82E9BR.3 influences animal physiology in a different way. It is more likely that ATPsyn-c (Y82E9BR.3), ATPsyn-b (*atp-2*) and ATPsyn-d (*atp-5*) affect ATP synthase and lifespan through different mechanisms. This also suggests that lifespan extension is not necessarily correlated with reduced ATP levels, as supported by studies using both *C. elegans* and Drosophila, which have shown that disruption of mitochondrial genes that extends longevity does not necessarily impact ATP levels^11,19,55^.

Impaired mitochondrial respiration elicits retrograde signalling in *C. elegans*^4,28^. In our study, worms treated with Y82E9BR.3 RNAi displayed an increased mtDNA levels, consistent with the previous observations using *clk-1* and *isp-1* mutants^28^. Our data further support the possible notion that animals with the defects in mitochondrial respiration initiate a compensatory response to produce more mitochondria.

ATP synthase is highly conserved and widely distributed in bacteria, eukaryotic mitochondria and chloroplasts. The C subunit functions in H^+^ transport, and structurally possesses a conserved acidic residue in the transmembrane helix, but differs in the surrounding residues among species^56^, potentially serving as a drug target for certain chemicals^57^. Multiple deficiencies in the subunits of ATP synthase have been reported in patients. In particular, a patient with conditions such as neonatal onset, lactic acidosis, 3-methylglutaconic aciduria and mild mental retardation has a mutation in ATP synthase subunit epsilon, with a remarkable accumulation of subunit c^58^. It was suggested that the compensatory responses to counteract mitochondrial mutations in the Mit mutants are very likely the same ones initiated to slow disease appearance in many human mitochondrial associated diseases (HMADs)^59^. Thus, the mechanism presented in our study may also be relevant to the search for potential preventative therapies for ATP synthase deficiency related to HMADs.

In conclusion, we explored the role of the mitochondrial respiratory chain gene Y82E9BR.3 in aging in this study for the first time. We demonstrated that RNAi targeting Y82E9BR.3 extends lifespan by reducing mitochondrial respiration as well as suppression of complex I activity. Our findings uncover the mechanism of the regulation of Y82E9BR.3 on lifespan and reveal the importance of the Y82E9BR.3 protein in determining aging and lifespan in *C. elegans*.

## Materials and Methods

### Strains

The following strains were analyzed in this study: N2 wild type, WM27 *rde-1(ne219) V*., NR222 *rde-1(ne219) V.; kzls9[lin-26p::nls::gfp; lin-26p::rde-1; rol-6D]*, NR350 *rde-1(ne219) V.; kzls20[hlh-1p::rde-1; sur-5p::nls::gfp]*, VP303 *rde-1(ne219) V.; kbls7[nhx-2p::rde-1; rol-6D]*, NL3321 *sid-1(pk3321) V., TU3401 sid-1(pk3321) V.; uIs69[myo-2p::mCherry; unc-119p::sid-1]*, JM43 *rde-1(ne219) V.; Is[wrt-2::RDE-1], and TK22 mev-1(kn1) III*.

### RNA interference

The Y82E9BR.3-specific RNAi clone, which was prepared previously^21^, was cultured overnight in LB broth containing 50 μg/ml ampicillin (USB, Santa Clara, CA, USA). To induce double-stranded RNA, 1 mM isopropyl β-D-1-thiogalactopyranoside (IPTG; Gold Biotechnology, St. Louis, MO, USA) was added to plates for incubation at room temperature for 1 day before usage.

### Quantitative RT-PCR

Approximately 500–1000 synchronized RNAi-treated young adult worms were used for quantitative RT-PCR analysis. Extraction, purification and reverse transcription of RNA were performed as previously described^51^. Quantitative PCR using the cDNA was executed in a StepOne Real Time PCR System (Applied Biosystems, Foster City, CA, USA) and analyzed using a comparative CT method. Average mRNA levels for *ama-1* (the large subunit of RNA polymerase II) were used for normalization. The average of two technical repeats was used for each biological data point.

Sequences of primers used for quantitative RT-PCR are listed below:

Y82E9BR.3-F-CCTCCTCGCCTCGAGAGCCCCACTC;

Y82E9BR.3-R-CGGCTCCAGCTCCGATGTACTTGGC;

*ama-1*-F-TGGAACTCTGGAGTCACACC;

*ama-1*-R-CATCCTCCTTCATTGAACGG.

### Brood size

Experiments were performed as described previously^60^. Single late-L4 stage worms were placed on individual RNAi-bacteria plates (10 worms per condition). The animals were transferred to new RNAi-bacteria-containing plates every 24 hours until they stopped producing progeny. Trials involving worms that bagged, ruptured, burrowed or crawled off were censored. All plates containing progeny were placed in a 20 °C incubator until the progeny reached L4 or adulthood, and the number of worms that developed was counted. Total brood size was determined by adding the numbers of progeny produced by a single worm each day.

### Lipofuscin accumulation

The autofluorescence of intestinal lipofuscin was measured as an index of senescence at day 18 of adulthood. Day-18 worms from the RNAi-bacteria NGM plates were picked and mounted onto a 2% agarose pad coated with 10 mM sodium azide (Junsei Chemical, Tokyo, Japan) in M9 buffer to induce anaesthesia. Lipofuscin autofluorescence images were captured using a DAPI (4′,6-diamidino-2-phenylindole) channel from an AxioCam HRc CCD digital camera (Zeiss Corporation) with a Zeiss Axio Scope A1 compound microscope (Zeiss Corporation). The fluorescence intensity of the animals was quantified by using ImageJ software (Rasband, W.S., ImageJ, U. S. National Institutes of Health, Bethesda, MD, USA, http://rsb.info.nih.gov/ij/).

### Swimming rate

Locomotion was measured as described previously with modifications^61^. Briefly, gravid adults were placed on plates containing RNAi bacteria to synchronize the worms, and the progeny were allowed to develop to young adults and were transferred onto RNAi plates containing 10 μM 5-fluoro-2′-deoxyuridine (FUdR, Sigma, St Louis, MO, USA), 50 μg/ml ampicillin (USB, Santa Clara, CA, USA), and 1 mM isopropyl β-D-1-thiogalactopyranoside (IPTG; Gold Biotechnology, St. Louis, MO, USA). To measure body bending in liquid, day-1 and day 9 worms were transferred into each well of the 24-well plates containing 1 ml of M9 buffer. After 30 seconds for equilibration, worm movements were recorded using a DIMIS-M (Siwon Optical Technology, Anyang, South Korea) camera, and the body bends of individual worms were counted for 30 seconds. The number of bend per minute were calculated.

### Feeding rate

Feeding rate measurements were performed as described previously with some modifications^61^. Briefly, gravid adults were placed on plates containing RNAi bacteria to synchronize the worms, and progeny were allowed to develop to young adults and were then transferred onto RNAi plates containing 10 μM 5-fluoro-2′-deoxyuridine (FUdR, Sigma, St Louis, MO, USA), 50 μg/ml ampicillin (USB, Santa Clara, CA, USA), and 1 mM isopropyl β-D-1-thiogalactopyranoside (IPTG; Gold Biotechnology, St. Louis, MO, USA). Pharyngeal pumping (feeding) rates were counted for 15 seconds by observing the pharynx of a day-1 and day 9 worm under a dissecting microscope using a Zeiss SteREO Discovery.V8 stereomicroscope (Zeiss, Germany), and converted to the number of pump per minute.

### Y82E9BR.3 promoter-driven GFP reporter

To generate the *Y82E9BR.3p::gfp* DNA construct, a DNA fragment that contained 2.0 kb upstream of the start codon of Y82E9BR.3 from *C. elegans* genomic DNA was PCR amplified. The DNA fragment was inserted into the pPD95.75 vector (Fire lab *C. elegans* vector kit), which contains *unc-54* 3′UTR and GFP and was constructed using the In-Fusion HD Cloning Kit (Clontech Laboratories, Inc, Mountain View, CA, USA) following the manufacturer’s instruction. The constructs were transformed into DH5a competent cells. The resulting plasmid (25 ng/μL) was microinjected into the gonad of day 1 adult worms with the coinjection marker *odr-1p::rfp* (75 ng/μL)^62^.

Transformed offspring were selected by picking the RFP-expressing F1 larvae. Stable transformants were picked among the F2 generation worms. In these transformants, the transcriptional activity of the Y82E9BR.3 promoter was reported by green fluorescence. The fluorescence and bright-field images were captured using an AxioCam HRc CCD digital camera (Zeiss Corporation) with a Zeiss Axio Scope A1 epifluorescence microscope (Zeiss Corporation). Images were merged using ImageJ software (Rasband, W.S., ImageJ, U. S. National Institutes of Health, Bethesda, Maryland, USA, http://rsb.info.nih.gov/ij/, 1997–2009).

### Generation of transgenic worms and fluorescence observation

*Y82E9BR.3p::Y82E9BR.3::gfp* transgenic animals were generated as described previously, with some modifications^50^. A promoter and the coding region of Y82E9BR.3 (2.4 kb) were cloned into the pPD95.75 vector using the In-Fusion HD Cloning Kit (Clontech Laboratories, Inc, Mountain View, CA, USA) following the manufacturer’s instructions. Constructs were transformed into DH5a competent cells. The construct was microinjected into the gonad of day 1 adult worms with the coinjection marker *odr-1p::rfp*. The transgenes showed an apparent genotoxicity in the process: the Y82E9BR.3::GFP fusion construct at an injection concentration of 25 ng/μl showed strong genotoxic effects, and we were not able to obtain transformants; the construct at 5 ng/μl showed decreased genotoxicity and we obtained only transient transformants; when the construct concentration decreased to 1 ng/μl, we acquired transmitted lines. For imaging of *Y82E9BR.3p::Y82E9BR.3::gfp* animals, animals were synchronized on OP50-seeded plates, and the fluorescence and bright field images were captured using a two-photon confocal fluorescence microscope (Leica TCS SP8 MP). Confocal images were obtained using a 488-nm excitation laser and GFP image was captured at the emission wavelength of 500–560 nm.

### Staining of mitochondria

MitoTracker staining was performed as described previously, with some modifications^63^. MitoTracker CMXRos (Invitrogen) was dissolved in dimethylsulfoxide (DMSO) to a stock concentration of 10 mM and was added to animals on the plates to a final concentration of 10 μM for 6 hours before analyses. The images were photographed by using the Nikon A1si/Ni-E upright confocal microscope with 100×, 1.4 NA oil-immersion objective lens. Confocal microscopy data were acquired at the Brain Research Core Facilities in Korea Brain Research Institute (KBRI).

### Silencing Y82E9BR.3 in Y82E9BR.3::GFP transgene

Y82E9BR.3p::Y82E9BR.3::GFP (IJ1392) worms were synchronized on the control or Y82E9BR.3 RNAi bacteria-seeded plates. When worms were reached L2 – L3 stages, the worms were anesthetized by using 100 mM sodium azide and imaged by using AxioCam HRc camera mounted on a Zeiss Axioscope A.1 microscope. The GFP intensity was quantified by using ImageJ software (Rasband, W.S., ImageJ, U. S. National Institutes of Health, Bethesda, MD, USA, http://rsb.info.nih.gov/ij/).

### Lifespan analysis

Lifespan assays were performed as described previously, with some modifications^64^. Briefly, RNAi clones were cultured overnight in LB with 50 μg/mL ampicillin (USB) at 37 °C and seeded onto nematode growth media (NGM) plates containing 50 μg/mL ampicillin. RNAi bacteria seeded on plates were treated with 1 mM isopropylthiogalactoside (IPTG; Gold Biotechnology, St. Louis, MO, USA) to induce dsRNA expression for 1 d at room temperature.

Gravid adults were placed on plates containing RNAi bacteria to synchronize the worms. The progeny were allowed to develop to young adults. Young adults were then transferred onto 5 μM 5-fluoro-2′-deoxyuridine (FUdR, Sigma, St. Louis, MO, USA)-treated RNAi-bacteria NGM plates; or young adult worms were transferred onto 5 μM FUdR (Sigma, St. Louis, MO, USA)-treated RNAi-bacteria NGM plates with or without 8 mM α-ketoglutarate (α-KG). Approximately 120 worms per condition were examined for death every 2 or 3 days until all animals were dead. Animals that ruptured, bagged, burrowed, or crawled off the plates were censored but used as censored subjects for statistical analysis. Lifespan assays were performed at 20 °C. Online application of survival analysis (OASIS; sbi.postech.ac.kr/oasis)^65^ was used for statistical analysis. *P* values were calculated using the log-rank (Mantel-Cox) method.

### Measurement of oxygen consumption rates

Oxygen consumption rates were measured using the Oxytherm (Hansatech; Norfolk, UK), a Clark-type oxygen electrode as described previously^60^. Briefly, approximately 5000 synchronized day 1 adult worms were collected, quickly washed three times in M9 buffer, resuspended in 1 ml of M9, and transferred into the chamber. Oxygen concentration was monitored with a Clark electrode in a closed chamber for ~10 min. Worms were collected, pelleted, and kept at −80 °C for protein quantification using a Bradford Protein Assay kit (Bio-Rad). Oxygen consumption rates were normalized to protein contents. The experiments were repeated five times.

### ATP detection

ATP levels were quantified using the luciferin-luciferase method based ATP Determination Kit (Invitrogen, Carlsbad, USA)^40^. Briefly, synchronized young adult worms fed on Y82E9BR.3 RNAi bacteria or control RNAi bacteria were collected and washed three times in M9 buffer. The worm pellets were treated with three freeze/thaw cycles and boiled for 15 min to release ATP and destroy ATPase activity. Worms were centrifuged at 12,000 × g for 10 min at 4 °C. Aliquots of the supernatant were used for the ATP assay following the manufacturer’s instructions. Luminescence was measured with a microplate reader (Infinite M200PRO, TECAN). The total protein concentration of the samples was determined using Bradford (Sigma, USA) assay. ATP content was normalized against the protein level. Three independent experiments were performed.

### Preparation of mitochondria

Mitochondria were isolated following the previously described method^66^. Briefly, wild type worms were fed Y82E9BR.3 RNAi bacteria or control RNAi bacteria, and *mev-1(kn1)* worms were cultured on NGM plates until reaching a young adult stage. Approximately 300,000 age-synchronized animals were harvested from the cultures, cleaned and washed with M9 buffer and suspended in mitochondrial isolation buffer (MSME)^67^. All subsequent treatments were performed at 4 °C. The worm suspension was ruptured using a homogenizer (IKA, Staufen, Germany). An equal volume of MSME containing 0.4% bovine serum albumin (BSA) was added, and the suspension was thoroughly mixed and centrifuged for 5 min at 380 × g. The supernatant was centrifuged repeatedly. The resulting supernatant containing crude mitochondria was centrifuged for 5 min at 4500 × g, and the resulting mitochondrial pellet was resuspended in MSME. Aliquots were used to determine total protein contents using the Bradford (Sigma, USA) assay. The remainder was frozen at −80 °C for quantification of mitochondrial respiratory chain (MRC) enzymatic activities.

### MRC enzymatic activity determination

The activities of the following MRC enzymes from the isolated mitochondria were assayed spectrophotometrically using a Mitochondria Complex I-V Activity Assay Kit (Genmed Scientifics)^68^: rotenone-sensitive NADH-ubiquinone oxidoreductase (complex I), succinate dehydrogenase (complex II), antimycin A-sensitive decylubiquinol cytochrome c oxidoreductase (complex III), cytochrome c oxidase (complex IV) and ATP synthase (complex V). Briefly, frozen (−80 °C) samples of mitochondria preparations were thawed on ice and solubilized with 2% CHAPS, and aliquots were used for enzymatic activity assay following the protocol of the manufacturer. The reactions used for the determination of complexes I-V were monitored at 340, 600, 550, 550 and 340 nm using a spectrophotometer (SHIMADZU 2450, Japan) respectively, and the activities of complexes I-V were calculated according to the formula given in the protocols. The activities of Complex I-V were normalized to total protein contents, measured as described above, and reported in nmol substrate/min/mg protein. Six independent experiments were performed.

### mtDNA content quantification

mtDNA contents were measured as described previously with some modifications^28^. Synchronized worms were cultured on the corresponding RNAi plates until reaching young adult stage. Single worm was lysed by using proteinase K. qPCR reaction was performed with SYBR green. Mito1 primer set was used for the mtDNA measurement. Sequences of primers used for quantitative RT-PCR are listed below:

Mito-1-F-GTTTATGCTGCTGTAGCGTG;

Mito-1-R-CTGTTAAAGCAAGTGGACGAG.

*ama-1*-F-TGGAACTCTGGAGTCACACC;

*ama-1*-R-CATCCTCCTTCATTGAACGG.

## Electronic supplementary material

Supplementary information

## Data Availability

The datasets generated during and/or analysed during the current study are available from the corresponding author on reasonable request.
