# Supplementary material for: Genetic inhibition of an ATP synthase subunit extends lifespan in C. elegans
Source: Sci Rep. 2018 Oct 4;8:14836. doi: 10.1038/s41598-018-32025-w (PMC6172204; doi:10.1038/s41598-018-32025-w)
Supplement: Supplementary file 1 — Supplementary information [file 41598_2018_32025_MOESM1_ESM.pdf]

Supplementary Information

Genetic inhibition of an ATP synthase subunit extends lifespan in *C. elegans*

Chen Xu[1,*], Wooseon Hwang[2,*], Dae-Eun Jeong[2], Youngjae Ryu[5], Chang Man Ha[5], Seung-Jae V Lee[2,3,4], Lulu Liu[1] and Zhi Ming He[1]

[1] State Key Laboratory of Pharmaceutical Biotechnology, School of Life Sciences, Nanjing University, Nanjing 210023, China.

[2] Department of Life Sciences, Pohang University of Science and Technology, Pohang, Gyeongbuk 37673, South Korea.

[3] Department of IT Convergence and Engineering, Pohang University of Science and Technology, Pohang, Gyeongbuk 37673, South Korea.

[4] School of Interdisciplinary Bioscience and Bioengineering, Pohang University of Science and Technology, Pohang, Gyeongbuk 37673, South Korea.

[5] Brain Research Core Facilities, Korea Brain Research Institute, Daegu 41068, South Korea.

*These authors contributed equally to this work.

Correspondence and requests for materials should be addressed to C.X. (email: xuchn@nju.edu.cn)

Supplementary Table

Table S1. Analysis of lifespan assay results.

**Strain/treatment**	**Mean lifespan ±s.e.m. (days)**	**75th percentile**	**% change**	**Number of animals that died/total**	***p* value vs. control**	**Figure in text**
WT control RNAi	16.49±0.48	19		93/121		Fig. 1A
Y82E9BR.3 RNAi	26.81±0.77	32	62.58%	88/120	<0.0001	
WT control RNAi	19.83±0.60	25		88/120		
Y82E9BR.3 RNAi	28.68±0.85	33	44.63%	44/120	<0.0001	
rde-1(ne219) Control RNAi	13.8±0.17	17		102/120		Fig. 3A
Y82E9BR.3 RNAi	14.53±0.18	17	5%	108/120	0.0050	
rde-1(ne219) Control RNAi	20.68±0.4	25		102/120		
Y82E9BR.3 RNAi	19.76±0.33	25	-4%	115/120	0.0263	
rde-1(ne219); kbls7[nhx-2p::rde-1; rol-6D] Control RNAi	16.57±0.32	19		92/120		Fig. 3B
Y82E9BR.3 RNAi	21.32±0.62	27	29%	95/120	<0.0001	
rde-1(ne219); kbls7[nhx-2p::rde-1; rol-6D] Control RNAi	20.72±0.52	25		98/120		

Y82E9BR.3 RNAi	24.91±0.63	30	20%	85/120	<0.0001	
rde-1(ne219); kzls9[lin-26p::nls::gfp; lin-26p::rde-1; rol-6D] Control RNAi	19.48±0.45	23		93/122		Fig. 3C
Y82E9BR.3 RNAi	18.41±0.37	21	-5%	101/120	0.0217	
rde-1(ne219); kzls9[lin-26p::nls::gfp; lin-26p::rde-1; rol-6D] Control RNAi	25.35±0.49	28		86/120		
Y82E9BR.3 RNAi	23.91±0.48	28	-6%	110/120	0.0365	
rde-1(ne219); kzls20[hlh-1p::rde-1; sur-5p::nls::gfp] Control RNAi	14.63±0.31	17		92/117		Fig. 3D
Y82E9BR.3 RNAi	13.62±0.19	17	-7%	100/120	0.0040	
rde-1(ne219); kzls20[hlh-1p::rde-1; sur-5p::nls::gfp] Control RNAi	18.94±0.35	22		89/120		
Y82E9BR.3 RNAi	18.8±0.27	22	-1%	89/120	0.3037	
rde-1(ne219);Is[wrt-2::RDE-1] Control RNAi	15.83±0.44	19		93/120		Fig. 3E
Y82E9BR.3 RNAi	14.35±0.39	15	-9%	93/121	0.0091	
rde-1(ne219);Is[wrt-2::RDE-1] Control RNAi	21.38±0.48	25		88/120		

Y82E9BR.3 RNAi	19.66±0.48	24	-8%	100/120	0.0040	
sid-1(pk3321) Control RNAi	16.39±0.34	20		98/120		Fig. 3F
Y82E9BR.3 RNAi	19.64±0.43	22	20%	98/121	<0.0001	
sid-1(pk3321) Control RNAi	19.51±0.59	23		89/120		
Y82E9BR.3 RNAi	19.87±0.49	23	2%	88/120	0.8384	
sid-1(pk3321); uIs69 [myo-2p::mCherry; unc-119p::sid-1] control RNAi	17.42±0.41	20		108/120		Fig. 3G
Y82E9BR.3 RNAi	19.89±0.37	21	14%	106/120	0.0006	
sid-1(pk3321); uIs69 [myo-2p::mCherry; unc-119p::sid-1] control RNAi	17.96±0.35	20		102/120		
Y82E9BR.3 RNAi	21.88±0.46	25	22%	108/120	<0.0001	
WT control RNAi	18±0.49	22		75/120		Fig. 4F
control RNAi/α-KG	20.1±0.51	22	11.67%	98/120	0.0044	
Y82E9BR.3 RNAi	25.84±0.58	31	43.56%	106/120	<0.0001	
Y82E9BR.3 RNAi/α-KG	22.56±0.52	26	-12.69%	87/120	<0.0001 Y82E9BR.3 RNAi	
WT control RNAi	16.54±0.47	19		93/120		
control RNAi/α-KG	18.07±0.5	21	9.25%	97/120	0.0329	

Y82E9BR.3 RNAi	26.14±0.55	30	58.04%	78/120	<0.0001	
Y82E9BR.3 RNAi/α-KG	23.04±0.54	26	-11.86%	73/120	0.0002 Y82E9BR.3 RNAi	
WT control RNAi w/o FUdR	19.83±0.6	25		88/120		Fig. S2
Y82E9BR.3 RNAi w/o FUdR	28.68±0.85	33	44.63%	40/90	<0.0001	
WT control RNAi w/o FUdR	15.98±0.36	19		91/120		
Y82E9BR.3 RNAi w/o FUdR	27.64±0.56	33	73.0%	81/117	<0.0001	

In this table, the survival data sets within solid lines were done in parallel and statistical analysis was done within the sets. Differences in conditions were distinguished by dotted lines. *p* values were calculated within the sets, and were calculated by using the log-rank (Mantel-Cox) method. Percent changes of mean lifespan of control RNAi-treated mutants were calculated against those of control RNAi-treated wild-type worms. Percent changes of mean lifespan of RNAi-treated wild-type and mutant animals were calculated against control RNAi-treated wild-type and mutant worms, respectively. *p* values for each condition were calculated against the control immediately above.

Supplementary Figure Legends

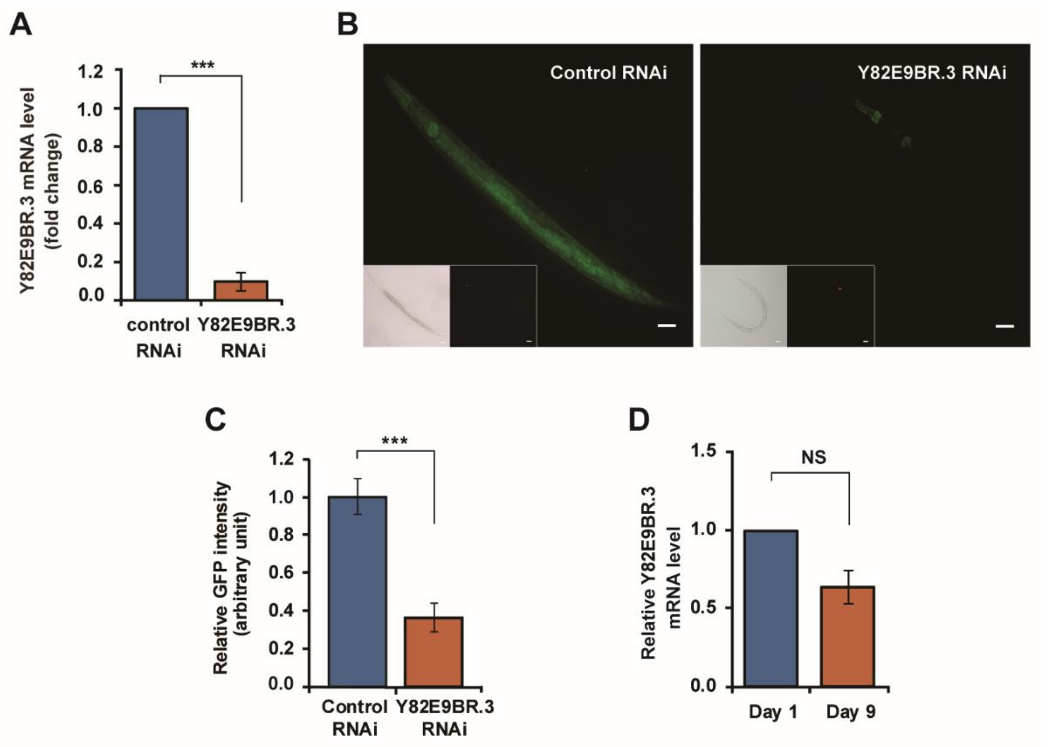

Figure S1. Y82E9BR.3 mRNA and Y82E9BR.3 Silencing in *C. elegans*. (**A**) Y82E9BR.3 mRNA expression significantly decreased in worms fed with Y82E9BR.3 RNAi. Y82E9BR.3 mRNA level was quantified using real-time PCR. Experiments were repeated three times. Error bars represent SEM (*$P < 0.05$, **$P < 0.01$, ***$P < 0.001$, two-tailed Student's *t*-test). (**B-C**) Feeding Y82E9BR.3 RNAi silence target gene in Y82E9BR.3::GFP transgenic worms. (B) Representative images of an L2 larval animal expressed Y82E9BR.3::GFP fed with control or Y82E9BR.3 RNAi. Expression of GFP was observed in the pharynx, intestine and hypodermis in control RNAi fed worm (*left*), but GFP expression was only observed in the pharynx in

Y82E9BR.3 RNAi fed worm (*right*). Y82E9BR.3 RNAi successfully knockdown the Y82E9BR.3 expression in almost all the tissues including the intestine. Red channel showing specific expression of coinjection marker *odr-1p::rfp* in the pharynx. Insets: bright-field and red channel images. Scale bar indicates 100 µm. (C) GFP fluorescence on panel (B) was quantified by using ImageJ. (Two trials, n=29). Error bars represent SEM (***$P < 0.001$, two-tailed Student's *t*-test). (**D**) Y82E9BR.3 mRNA expression in young (Day 1) and old (Day 9) wild type worms was quantified using real-time PCR. Experiments were repeated four times. Error bars represent SEM (two-tailed Student's *t*-test).

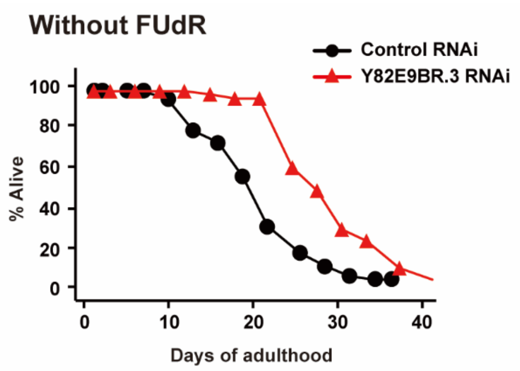

Figure S2. The lifespan of *C. elegans* fed with Y82E9BR.3 RNAi without FUdR. Y82E9BR.3 RNAi extended the lifespan of wild-type animals without FUdR treatment. See supplementary table S1 for statistical analysis and additional repeats.
